# Supplementary material for: Association of UBE3C Variants with Reduced Kidney Function in Patients with Diabetic Kidney Disease
Source: J Pers Med. 2020 Nov 6;10(4):210. doi: 10.3390/jpm10040210 (PMC7712123; doi:10.3390/jpm10040210)
Supplement: Supplementary file 1 [file jpm-10-00210-s001.pdf]

Supplementary Material

Table S1. Baseline characteristics of the microarray study population.

**Characteristics**	**High UPCR**	**Low UPCR**
Total Number	4	4
Male, n (%)	4 (100)	4 (100)
Age (years)[a]	67 ± 10.55	65.25 ± 15.92
BMI (kg/m^2)[a]	26.41 ± 2.76	21.46 ± 3.14
Smoking, n (%)	1 (25)	0 (0)
HbA1c (%)[a]	6.55 ± 0.58	8.73 ± 3.12
eGFR (ml/min/1.73 m^2)[a]	26.20 ± 18.77	35.93 ± 9.46
UPCR (mg/g)[a]	7048.90 ± 2900.06	646.83 ± 227.25

[a] Mean ± standard deviation

Abbreviations: BMI, body mass index; HbA1C, hemoglobin A1c; eGFR, estimated glomerular filtration rate; UPCR, urine protein and creatinine ratio.

Table S2. The list of oppositely regulated, significant DEGs between patients with high UPCR and patients with low UPCR.

Gene name	*p*-value [a]	ln FC [a]
UBE3C	0.001198	-0.56939
TCEAL4	0.001503	0.58648
PRSS23	0.002540	-0.89225
CD8B	0.002798	-1.16020
MRPL48	0.002815	-0.54051
LAIR2	0.002943	-1.08743
ADRB2	0.004139	-0.55549
SWAP70	0.006187	0.55440
SLC39A8	0.006672	-0.51289
TLE4	0.008125	0.54956

[a] p value < 0.01 and |ln FC| >0.5 were considered to be significant.

Abbreviations: DEGs, differentially expressed genes; FC, fold change; UCPR, urine protein to creatinine ratio.

Table S3. Results of the association analysis between *UBE3C* SNPs and diabetic kidney disease susceptibility in the case group (patients with a UPCR of >150 mg/g) and the control group (patients with a UPCR of ≤ 150 mg/g).

		Number			Recessive Model	
SNP	Genotype	Case (%)	Control (%)	OR (95% CI)	*p* value	*q* value
rs3802129	G/G-A/G	143 (80.3)	61 (80.3)	1.00	0.988	0.998
	A/A	35 (19.7)	15 (19.7)	1.01 (0.51 - 1.99)		
rs3815217	A/A-A/G	131 (79.4)	55 (79.7)	1.00	0.998	0.998
	G/G	34 (20.6)	14 (20.3)	1.00 (0.50 - 2.02)		
rs6979947	A/A-A/G	139 (80.3)	60 (81.1)	1.00	0.897	0.998
	G/G	34 (19.7)	14 (18.9)	1.05 (0.52 - 2.11)		
rs12669987	C/C-C/T	159 (96.4)	69 (94.5)	1.00	0.49	0.998
	T/T	6 (3.6)	4 (5.5)	0.62 (0.17 - 2.33)		
rs8101	C/C-C/T	132 (74.6)	52 (72.2)	1.00	0.688	0.998
	T/T	45 (25.4)	20 (27.8)	0.88 (0.47 - 1.64)		
rs7807	A/A-A/C	150 (84.3)	64 (87.7)	1.00	0.565	0.998
	C/C	28 (15.7)	9 (12.3)	1.27 (0.56 - 2.86)		

The *p* values were adjusted for age and gender.
Abbreviations: OR, odds ratio; SNP, single-nucleotide polymorphisms; UPCR, urine protein-to-creatinine ratio.
